# Microarray analysis of Long non-coding RNA expression profiles in human gastric cells and tissues with *Helicobacter pylori* Infection

**DOI:** 10.1186/s12920-015-0159-0

**Published:** 2015-12-21

**Authors:** Hong Zhu, Qiang Wang, Yizheng Yao, Jian Fang, Fengying Sun, Ying Ni, Yixin Shen, Hua Wang, Shihe Shao

**Affiliations:** School of Medicine, Jiangsu University, 301 Xuefu Road, Zhenjiang, Jiangsu 212013 China; Department of Gastroenterology, The Second People’s Hospital of Changzhou, Changzhou, Jiangsu 213003 China

**Keywords:** Long non-coding RNAs, *Helicobacter pylori*, Microarray, Expression profile

## Abstract

**Background:**

Although *Helicobacter pylori* (*H.pylori*) is the dominant gastrointestinal pathogen, the genetic and molecular mechanisms underlying *H.pylori*-related diseases have not been fully elucidated. Long non-coding RNAs (lncRNAs) have been identified in eukaryotic cells, many of which play important roles in regulating biological processes and pathogenesis. However, the expression changes of lncRNAs in human infected by *H.pylori* have been rarely reported. This study aimed to identify the dysregulated lncRNAs in human gastric epithelial cells and tissues infected with *H.pylori*.

**Methods:**

The aberrant expression profiles of lncRNAs and mRNAs in GES-1 cells with or without *H.pylori* infection were explored by microarray analysis. LncRNA-mRNA co-expression network was constructed based on Pearson correlation analysis. Gene Ontology (GO) and KEGG Pathway analyses of aberrantly expressed mRNAs were performed to identify the related biological functions and pathologic pathways. The expression changes of target lncRNAs were validated by qRT-PCR to confirm the microarray data in both cells and clinical specimens.

**Results:**

Three hundred three lncRNAs and 565 mRNAs were identified as aberrantly expressed transcripts (≥2 or ≤0.5-fold change, *P* < 0.05) in cells with *H.pylori* infection compared to controls. LncRNA-mRNA co-expression network showed the core lncRNAs/mRNAs which might play important roles in *H.pylori*-related pathogenesis. GO and KEGG analyses have indicated that the functions of aberrantly expressed mRNAs in *H.pylori* infection were related closely with inflammation and carcinogenesis. QRT-PCR data confirmed the expression pattern of 8 (n345630, XLOC_004787, n378726, LINC00473, XLOC_005517, LINC00152, XLOC_13370, and n408024) lncRNAs in infected cells. Additionally, four down-regulated (n345630, XLOC_004787, n378726, and LINC00473) lncRNAs were verified in *H.pylori-*positive gastric samples.

**Conclusion:**

Our study provided a preliminary exploration of lncRNAs expression profiles in *H.pylori-*infected cells by microarray. These dysregulated lncRNAs might contribute to the pathological processes during *H.pylori* infection.

**Electronic supplementary material:**

The online version of this article (doi:10.1186/s12920-015-0159-0) contains supplementary material, which is available to authorized users.

## Background

*Helicobacter pylori* (*H.pylori*) is a human-specific gastric bacterium that colonizes more than half of the world’s population. The infection of this pathogen is thought to be persisted for lifetime without treatment. The pathogen can evoke both innate and adaptive immune responses. However, immune system fails to eradicate this causative agent, which can lead to the gastric mucosa pathological changes [1]. The clinical outcome, ranging from chronic gastritis to peptic ulcers, even to cancer or mucosa-associated lymphoid tissue (MALT) lymphoma, is caused by multiple interior genetic dysregulation in combination with various environmental factors and bacterial virulence factors [2, 3].

Long noncoding RNAs (lncRNAs) are a little-understood class of transcribed RNA molecules, exceeding 200 nucleotides in length but have no significant protein-coding capacity, which are identified as key regulators in various biological functions.[4]. It has been known that lncRNAs are linked to epigenetic regulation, body development, and physiological responses [5–7]. Moreover, recently evidences have revealed that remarkably expressed lncRNAs are correlated with human diseases, such as neurological disorders, cardiovascular diseases, autoimmune diseases, inflammatory diseases, infectious diseases and various cancers [8, 9]. The discovery of lncRNAs and investigation on their functions in regulatory networks could lead us to a deeper comprehension of pathogenesis. Recently, the researchers have made some achievements in understanding lncRNAs. However, the expression patterns and functions of lncRNAs in cells infected by *H. pylori* have been seldom reported. Considering the wide range of roles that lncRNAs play in cellular and molecular regulatory processes, we do believe that it is possible that the aberrant expression of some lncRNAs might contribute to the *H.pylori-*infection associated disorders and diseases.

In this study, we identified the expression patterns of lncRNAs in *H.pylori*-infected gastric epithelial cell GES-1 via microarray analysis. LncRNA-mRNA co-expression networks were built based on Pearson correlation analysis. Gene Ontology (GO) and Kyoto Encyclopedia of Genes and Genomes (KEGG) Pathway analysis were performed to analyze lncRNA related coding genes involved in distinguishable biological responses. Several aberrantly expressed lncRNAs were confirmed by qRT-PCR in cells and gastric mucosa tissues. Our results suggest that the aberrantly expressed lncRNAs might expand our understanding of pathogenesis in *H.pylori* related diseases.

## Methods

### Cell lines and cultures

Cell lines were purchased from the Institute of Biochemistry and Cell Biology, SIBS, CAS (Shanghai, China). The human normal gastric epithelial cell line GES-1 was routinely cultured in RPMI 1640 (Gibco, Grand Island, N.Y., USA). Human gastric cancer cell lines SGC-7901 and BGC-823 were propagated in low-glucose DMEM (Gibco). All the media were supplemented with 10 % (vol/vol) fetal bovine serum (FBS; Wisent Inc., Quebec, Canada). Cells were maintained in humidified air with 5 % CO_2_ at 37 °C before use.

### *H. pylori* culture and infection model

*H.pylori* wild-type strain 26695 was obtained from ATCC and cultured on Columbia Agar (OXOID, UK) plates containing 5 % FBS (Wisent Inc.) grown at 37 °C in a microaerophilic atmosphere for 48 to 72 h.

Cells were seeded in six-well cell culture plates before infection. The following day, 80 % confluent monolayer cells were washed in PBS, and the medium was replaced with fresh medium. Then, *H.pylori* was added to cells for 24 h at a multiplicity of infection (MOI) of 100 as experimental groups. Cells without *H.pylori* infection were maintained for indicated time periods as control groups. After 24h infection, cells were washed in PBS to wash away the non-adherent *H.pylori*, then harvested in TransZol Up reagent (TransGen Biotech, Beijing, China) and preserved at -80 °C until RNA isolation.

### RNA isolation

Total RNA was extracted from cells and tissues using TransZol Up reagent (TransGen Biotech) according to the manufacturer’s instructions. RNA quantification and purity were measured by ND-1000 spectrophotometer (NanoDrop Technologies, Inc., Wilmington, DE, USA) measuring absorbance ratios of A_260_/A_280_ and A_260_/A_230_, and RNA integrity assessed by standard denaturing agarose gel electrophoresis.

### Microarray analysis

The Human Transcriptome Array 2.0 (HTA 2.0; manufactured by Affymetrix Inc., Santa Clara, CA, USA) was employed in this study. HTA 2.0 covers global profiling of full-length transcripts, containing more than 40,000 non-coding and 245,000 coding transcripts in human genome; each transcript is accurately identified by specific exon or exon-exon splice junction probes. All of the transcripts were collected from multiple public sources such as NCBI RefSeq, Ensembl, UCSC (known genes and lincRNA transcripts), Vertebrate Genome Annotation (Vega) database, Mammalian Gene Collection (MGC) (v10), www.noncode.org, lncRNA db, Broad Institute, Human Body Map lincRNAs and TUCP catalog. In the cases of small samples, random variance model (RVM) *t*-test was applied to filter the aberrantly expressed transcripts for experimental and control group. After significant analysis and false discovery rate (FDR) analysis, the differentially expressed transcripts were selected according to the *P* < 0.05.[10–12]. The cDNA labeling, microarray hybridization and bioinformatics analysis were performed by Genminix Informatics, Shanghai, China.

### Construction of the lncRNA-mRNA co-expression network

The lncRNA-mRNA co-expression network was built according to the normalized signal intensity of specific expression lncRNAs or mRNAs. The Pearson correlation was calculated for each pair of lncRNA-mRNA, and then, the significant correlation pairs were chosen to construct the network [13, 14]. The co-expression network was built in *H.pylori*-infected group and control group, respectively. Within a network, “degree” is the simplest and most important measure of the centrality of one gene or lncRNA that determining the relative importance. The “degree” is defined as the link number of one transcript directly had to the other [15]. The “degree” in infected group was recorded as S_Degree, while in control group was recorded as C_Degree. In addition, “clustering coefficient” is a measure of the “degree” to which transcripts in a network tend to cluster together. It was calculated by the local measure [16]. To exclude other transcripts’ impact in each co-expression network, we further performed normalization of the “degree”, i.e., divided by the maximum value of the transcript degree in each network [Normalized degree(X) = degree(X)/degree(Max)]. Then, the difference value of a transcript’s normalized degree (delta normalized degree, represented as |DiffK|) was calculated between the experimental and control co-expression networks. The core lncRNA/mRNA always owned the largest |DiffK|s [17].

### Coding gene functional analysis

Gene Ontology (GO) and KEGG Pathway analysis were performed to clarify the function and biological pathways of differentially expressed lncRNA co-expressed mRNAs from our microarray data. The differentially expressed mRNAs were annotated according to their attributes of gene products. Gene Ontology (http://www.geneontology.org) was then used to assign the genes to different GO terms of their associated aspects: biological processes, cellular components and molecular functions, according to their annotations [18]. Furthermore, the biological function of genes can be better understood via integrated analysis of KEGG (http://www.genome.ad.jp/kegg/) Pathways and gene annotations [19, 20]. The *P*-value was used to determine the significance of the enrichment, and the false discovery rate (FDR) was used to evaluate the significance of the *P*-value. The significant GO terms and pathways were filtered in accordance with *P* < 0.05 and FDR < 0.05.

### Patient, specimens, and clinical data collection

The clinical specimens were collected from 126 patients with gastritis or ulcer at the Digestive Endoscopy center, and the gastric cancer tissues were collected from 30 gastric cancer patients undergoing surgery at the Second People’s Hospital of Changzhou in Changzhou, Jiangsu, China. In this study, 67 cases were *H.pylori*–positive, median age of patients (35 men and 32 women) was 49 years (range 20–81 years); 89 cases were *H.pylori*–negative as healthy control, median age of patients (45 men and 44 women) was 56 years (range 19–77 years). Three biopsy specimens taken from each patient were for histopathological examination, Rapid Urease Test (RUT; Huitai Medical tech corp., Shanghai, China), and for RNA extraction. The specimens were preserved in TransZol Up reagent at -80 °C until RNA isolation.

The *H.pylori* infection status was assessed by RUT and *H. pylori*-specific ureC polymerase chain reaction (PCR) [21]. *H.pylori*-positive patients were grouped under at least one of the tests yielded positive results. The patients neither received nonsteroidal anti-inflammatory drugs (NSAIDs) nor had taken antibiotics or proton pump inhibitor in the preceding 4 weeks. Informed consents were obtained from all individual participants enrolled in this study before examination. The information of specimens is presented in Additional file 1.

### Quantitative real-time PCR (qRT-PCR)

Following RNA extraction, 1 μg of RNA samples were reverse transcribed into cDNA using HiFiScript 1st Strand cDNA Synthesis Kit (CWBIOTECH, Beijing, China) according to the manufacturer’s protocols. The differentially expressed candidate lncRNAs in this study were verified by qRT-PCR (Table 1). Each qRT-PCR was performed using 2 × SYBR Green mix (TransGen Biotech) with cycling conditions of 94 °C for 5 min followed by 45 cycles of 94 °C for 30s, 58 or 60 °C for 25 s. For each sample, we performed qRT-PCR for target genes and a housekeeping gene *β*-actin as an internal control. The sequences of specific primers are listed in Table 2. After PCR amplification, melt curve analysis was performed to confirm reaction specificity; expression fold change of each lncRNA was calculated via the 2^-ΔΔCt^ method. Differences in expression levels between *H.pylori*–positive and negative samples were analyzed using Student’s *t*-test, with a value of *P* < 0.05 considered statistically significant.

**Table 1:** Microarray expression results of selected lncRNAs. “S” represents *H.pylori-*infected GES-1 groups, and “C” represents control groups. *P-*value < 0.05 was considered statistically significant

lncRNA Accession Number	Gene Symbol	Variation trend	Fold change (S/C)	*P*-value	lncRNA Source	Probe ID
n345630		down	0.099	<1e-07	NONCODE	TC04001940.hg.1
TCONS_00010304-XLOC_004787		down	0.14	< 1e-07	Rinn lincRNAs	TC05002959.hg.1
n378726		down	0.15	< 1e-07	NONCODE	TC06003993.hg.1
NR_026860	LINC00473	down	0.16	< 1e-07	RefSeq	TC06002302.hg.1
n345729		down	0.18	< 1e-07	NONCODE	TC05002683.hg.1
n342056		down	0.41	1.06E-05	NONCODE	TC12002644.hg.1
n384667		down	0.42	1.80E-06	NONCODE	TC05003053.hg.1
TCONS_00011401-XLOC_005517		up	1.63	4.24E-05	Rinn lincRNAs	TC06003154.hg.1
NR_038366	HOTAIRM1	up	1.65	1.66E-05	RefSeq	TC07000165.hg.1
NR_024204	LINC00152	up	2.11	1.00E-07	RefSeq	TC02000535.hg.1
NR_015379	UCA1	up	2.15	1.70E-06	RefSeq	TC19000279.hg.1
TCONS_00027385-XLOC_013370		up	2.22	1.85E-04	Rinn lincRNAs	TC19002530.hg.1
n408024		up	4.84	< 1e-07	NONCODE	TC0X001624.hg.1

**Table 2:** Primers designed for qRT-PCR validation of candidate lncRNAs and *β*-actin

lncRNA Accession Number	Forward primer	Reverse primer	Product length (bp)	Tm (°C)
n345630	5'-TCCGTTGAACCTTCCACAGT-3'	5'-ACTCTGCTCCGTTCCACATT-3'	168	58
TCONS_00010304-XLOC_004787	5'-CTCAGGAAAGGAGTATAGAATG-3'	5'-GGTGCAAGGTATAGAGTGT-3'	104	58
n378726	5'-CCACAATGCAAACAACTGCT-3'	5'-GAAAGCTGCTCTGTGGTGAA -3'	161	60
NR_026860	5'-CTTGGTTGTGCGGGATTCT-3'	5'-GTCAGAAGGAGGAGCAGGTAG-3'	204	60
n345729	5'-AGGGTCATTTAGCCAGAAAGT-3	5'-GATAAACCCAGATGCCCTTGTAG-3'	144	58
n342056	5'-CAGGCTTATGGAGCGTTAAGAAT-3'	5'-CATCAGGGAGAAGTTATCAGGT-3'	177	58
n384667	5'-TGCCTGATAAGGTCACATACAC-3'	5'-CCAGGACATGCGATGAAGATTG-3'	123	58
TCONS_00011401-XLOC_005517	5'-TCCTGGGTCAAGCTGAGTATC-3'	5'-TGGAGTCTTACAAATCTTTTA-3'	131	58
NR_038366	5'-ACTCCGTGTTACTCATTCC-3'	5'-TTGCTTCTTCTTCTCCTCTT-3'	188	58
NR_024204	5'-GAATAACTGGGAGATGAAACAGG-3'	5'-CAACAGGTAGAGGTGCTGGA-3'	102	58
NR_015379	5'-TCCATTCAGACCGCCACTCAC-3'	5'-CAAGGTGCCAGTTAGCGTAT-3'	244	58
TCONS_00027385-XLOC_013370	5'-GGCTGTCTTAGAAGGATGAA-3'	5'-AATAGAGCTGGTTGACTGC-3'	129	58
n408024	5'-CGGAAGGTTACAGTCTCTAG-3'	5'-TGCTGTGTCCTCATTTATCA-3'	125	58
*β*-actin	5'-GATGACCCAGATCATGTTTGAG-3'	5'-AGGGCATACCCCTCGTAGAT-3'	159	58-60

### Statistical analysis

All results were expressed as means ± standard deviation (SD) of three independent experiments. Statistical significance of difference in the means between groups was analyzed using Student’s *t*-test with SPSS software (version 20.0 SPSS Inc., Chicago, IL, USA) and GraphPad Prism 6.0 (GraphPad Software Inc., La Jolla, CA, USA). *P-*value <0.05 was considered statistically significant.

## Results

### Analysis of aberrantly expressed lncRNAs and mRNAs

**Fig. 1:**
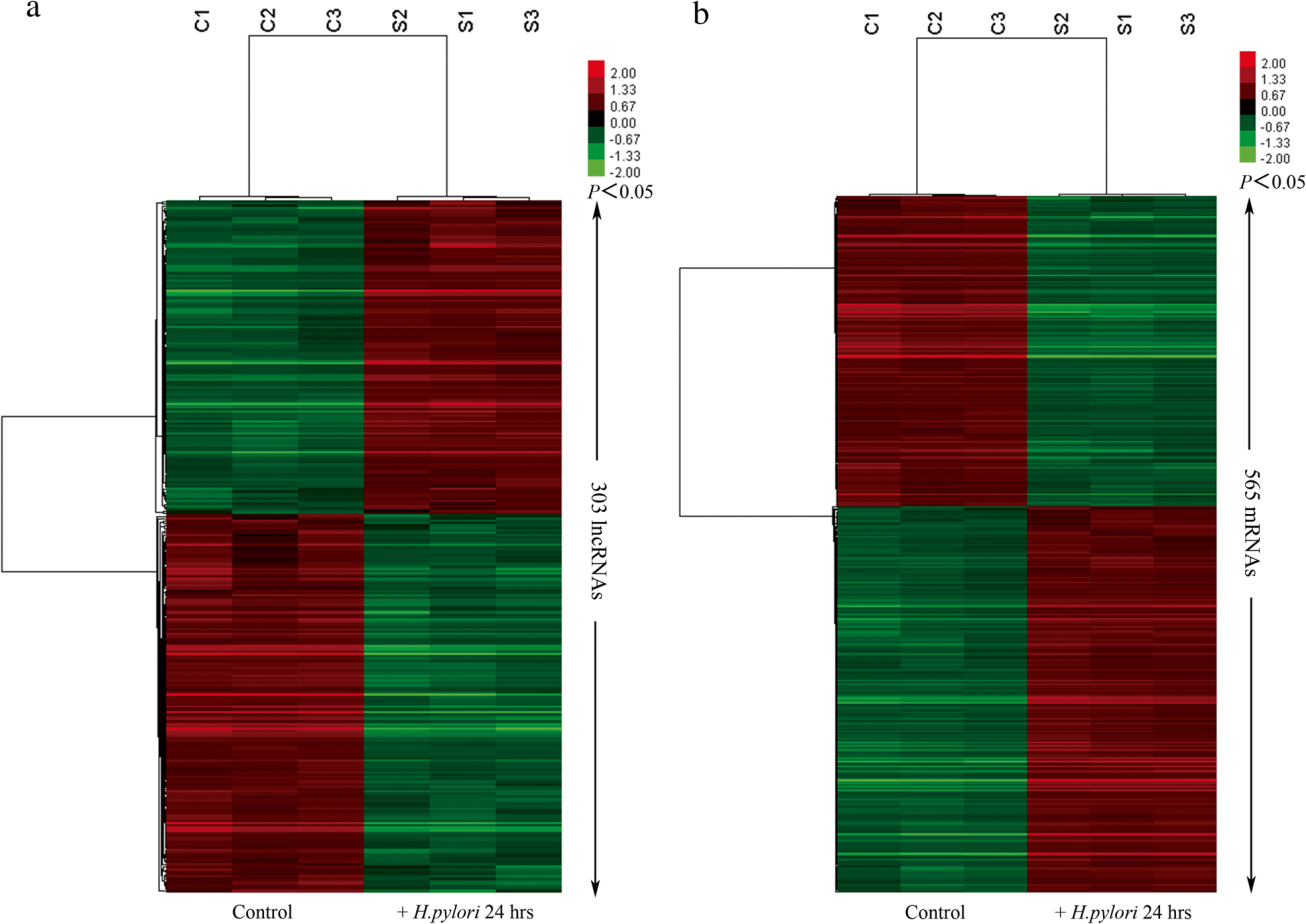
Hierarchical clustering analysis of gene expression levels between *H.pylori-*infected groups and control groups. 303 lncRNAs **a** and 565 mRNAs **b** were significantly changed in response to *H.pylori* infection. The clustering analysis was performed by Cluster 3.0 software with average linkage clustering algorithm and visualized by Treeview. “S” and “C” represent *H.pylori-*infected groups and control groups. “Red” and “green” indicate up-regulated and down-regulated transcripts, respectively

To explore the potential lncRNAs involved in *H.pylori*–induced gastric mucosa disorders, we examined the lncRNA and mRNA expression profiles in gastric epithelia cell models through microarray analysis (Fig. 1). According to the microarray data and the authoritative data, 303 unique lncRNAs were significantly induced or suppressed in GES-1 following *H.pylori* infection, of which 56.4 % (171 lncRNAs) were suppressed and 43.6 % (132 lncRNAs) were induced (fold change ≥2 or ≤0.5, *P* < 0.05) (Fig. 1a, Additional file 2). n335470 (11.48-fold change) was the most significantly up-regulated lncRNA and NR_002763 (CPS1-IT1, 0.075-fold change) was the most significantly down-regulated lncRNA.

As to mRNAs, the expression profiling data showed that of the total 1936 mRNAs, 565 were aberrantly expressed mRNAs in *H.pylori*-infection models relative to their control models (fold change ≥2 or ≤0.5, *P* < 0.05), of which 49.2 % (278 mRNAs) were up-regulated, while 50.8 % (287 mRNAs) were down-regulated (Fig. 1b, Additional file 2). Among these mRNAs, IGFBP1 (12-fold change) showed the highest degree of up-regulation, while MUC13 (0.11-fold change) was the most down-regulated protein-coding gene.

The complete microarray data are publicly available at Gene Expression Omnibus (GEO) database (http://www.ncbi.nlm.nih.gov/geo/) under the accession number GSE74577.

### LncRNA-mRNA co-expression network

We constructed the lncRNA-mRNA co-expression network to identify the interactions between mRNA and lncRNA. The value of “Degree” in co-expression network indicated that one mRNA/lncRNA might correlate with several lncRNAs/mRNAs. The core lncRNA/mRNA from the co-expression network were identified according to the |DiffK|s, and supposed to interact in processes of *H.pylori* infection. The lncRNAs with 30° in experimental group network are listed in Table 3, and the top 10 lncRNAs in co-expression network are presented in Table 4.

**Table 3:** The “Degree” of lncRNAs in co-expression network of experimental group (only no less than 30° is shown)

LncRNA	Type	Style	Degree	Clustering Coefficient
n336000	noncoding	up	32	0.8004
TCONS_00027385-XLOC_013370	noncoding	up	32	0.8004
n326267	noncoding	down	32	0.7903
n384365	noncoding	down	31	0.8215
n387010	noncoding	down	31	0.8215
n345751	noncoding	down	30	0.8253
NR_038407	noncoding	down	30	0.8069
n384667	noncoding	down	30	0.7701
n337099	noncoding	down	30	0.7563
n344793	noncoding	down	30	0.754

**Table 4:** The top ten lncRNAs with largest |DiffK| in co-expression network

LncRNA	Style	S_Degree	S_K	C_Degree	C_K	DiffK(S-C)	|DiffK|
n334184	up	26	0.7647	9	0.2432	0.5215	0.5215
NR_033917	down	11	0.3235	30	0.8108	−0.4873	0.4873
n339262	up	28	0.8235	13	0.3514	0.4722	0.4722
TCONS_00011401-XLOC_005517	up	27	0.7941	12	0.3243	0.4698	0.4698
n340399	down	9	0.2647	27	0.7297	−0.465	0.465
TCONS_l2_00005430-XLOC_l2_002852	down	12	0.3529	30	0.8108	−0.4579	0.4579
TCONS_00027385-XLOC_013370	up	32	0.9412	18	0.4865	0.4547	0.4547
n335665	up	28	0.8235	14	0.3784	0.4452	0.4452
n335607	up	21	0.6176	7	0.1892	0.4285	0.4285
TCONS_00010304-XLOC_004787	down	14	0.4118	31	0.8378	−0.4261	0.4261

### GO analysis and KEGG Pathway analysis of aberrantly expressed mRNAs

**Fig. 2:**
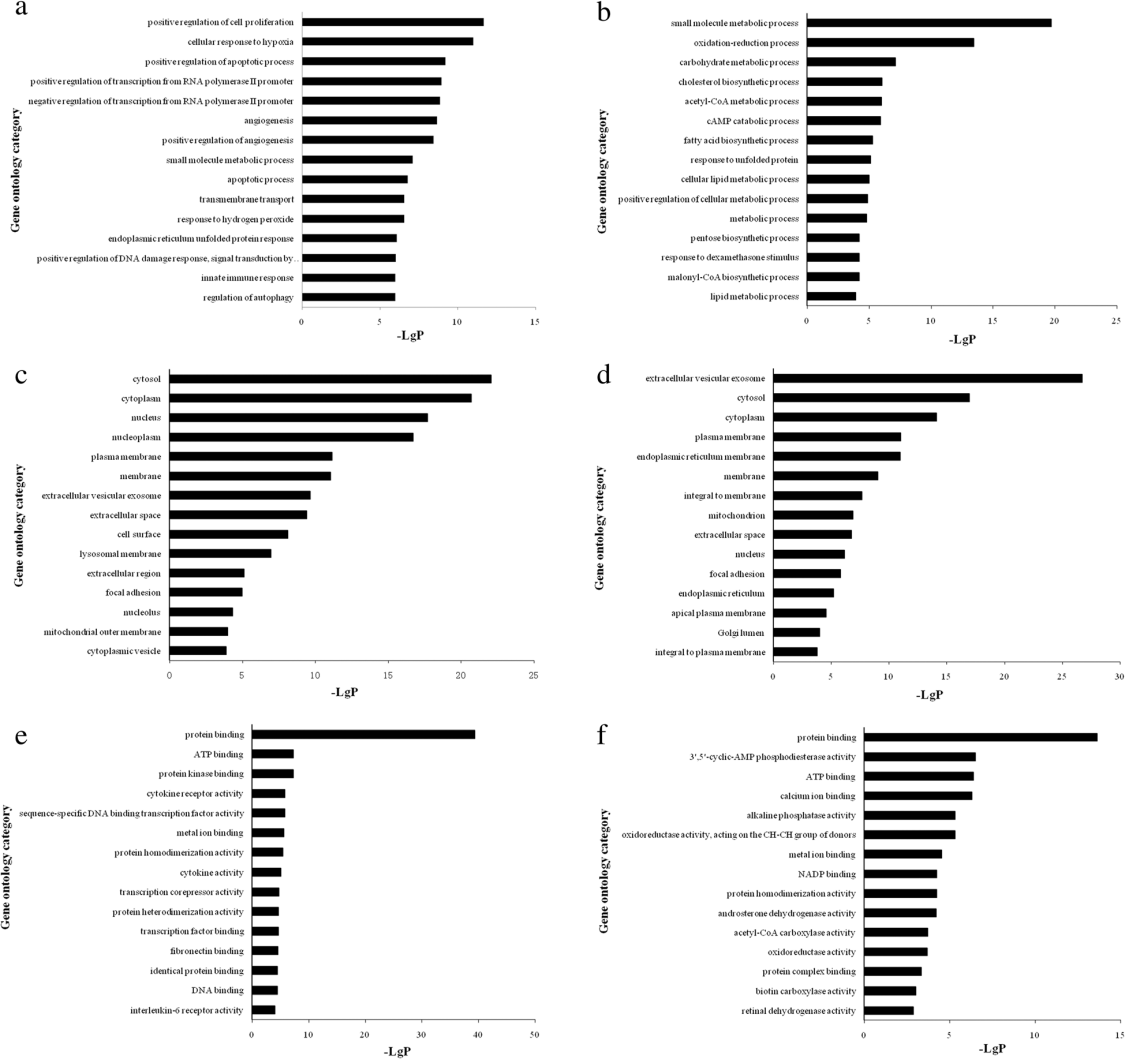
GO enrichment analysis of aberrantly expressed mRNAs. GO analysis of lncRNA co-expressed mRNAs according to biological process **a**, **b**, cell component **c**, **d** and molecular function **e**, **f. a**, **c**, **e**: the enriched GO terms targeted by up-regulated mRNAs; **b**, **d**, **f**: the enriched GO terms targeted by down-regulated mRNAs. The most enriched GO terms were filtered in accordance with *P* < 0.05 and FDR < 0.05. The bar plot “-Lg (*P*)” represents the enrichment score of enriched GO term

GO analysis was applied to investigate the potential functions of the lncRNAs co-expressed mRNAs on the regulation of the pathological responses against *H.pylori* infection. In this study, corresponding to the up-regulated mRNA, there were 189 aberrantly expressed mRNAs assigned to biological process, 213 assigned to cellular component and 181 assigned to molecular function; while among the down-regulated genes, there were 133 aberrant expressed mRNAs assigned to biological process, 170 assigned to cellular component and 137 assigned to molecular function. The significance of enrichment of each GO term was assessed by *P*-value <0.05 and FDR < 0.05, then the GO terms were filtered by the enrichment scores (-Lg (*P*)) in aberrantly expressed mRNAs. The enrichment analyses of top fifteen GO terms were listed in Additional file 3 and shown in Fig. 2. The GO enrichment analysis revealed that positive regulation of cell proliferation (GO: 0008284), cytosol (GO: 0005829), protein binding (GO: 0005515) were the most enriched GO terms targeted by aberrantly up-regulated mRNAs in biological process, cellular component and molecular function, respectively (Fig. 2 a, c, e). While the small molecule metabolic process (GO: 0044281), extracellular vesicular exosome (GO: 0070062) and protein binding (GO: 0005515) were the most enriched GO terms targeted by aberrantly down-regulated mRNAs in biological process, cellular component and molecular function, respectively (Fig. 2 b, d, f).

**Fig. 3:**
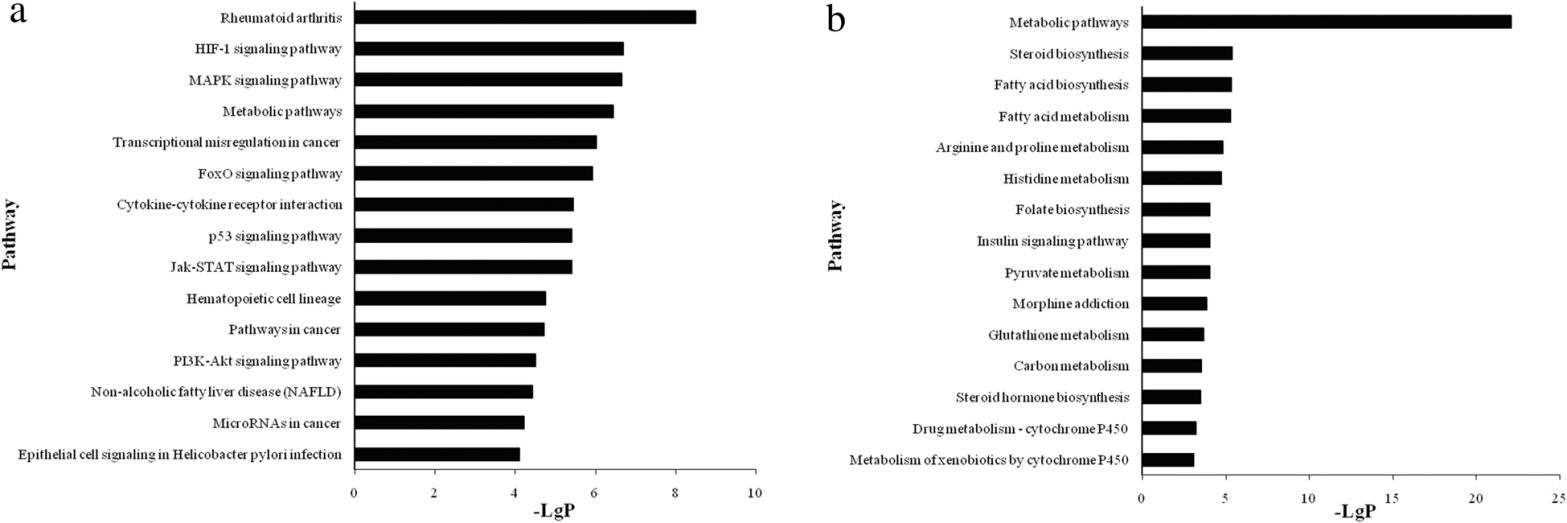
KEGG Pathway analysis of aberrantly expressed mRNAs. Top ten enriched up-regulated **a** and down-regulated pathways **b**. The most enriched pathways were filtered in accordance with *P* < 0.05 and FDR < 0.05. The bar plot “-Lg (*P*)” represents the enrichment score of enriched pathway

KEGG Pathway analysis offered us a reliable way to elucidate the candidate biological pathways that the lncRNAs interacted with mRNAs. We identified 54 up-regulated pathways comprising 90 differentially expressed genes, among them, the top three enriched pathways were rheumatoid arthritis (pathway ID: 05323), HIF-1 signaling pathway (pathway ID: 04066), and MAPK signaling pathway (pathway ID: 04010). 40 down-regulated pathways containing 61 differentially expressed genes were identified, and the top three enriched pathways were metabolic pathways (pathway ID: 01100), steroid biosynthesis (pathway ID: 00100), and fatty acid biosynthesis (pathway ID: 00061). The enrichment analyses of top fifteen pathways were showed in Fig. 3, the pathways and genes were listed in Additional file 4.

### Validation of the expression levels of lncRNAs by qRT-PCR

**Fig. 4:**
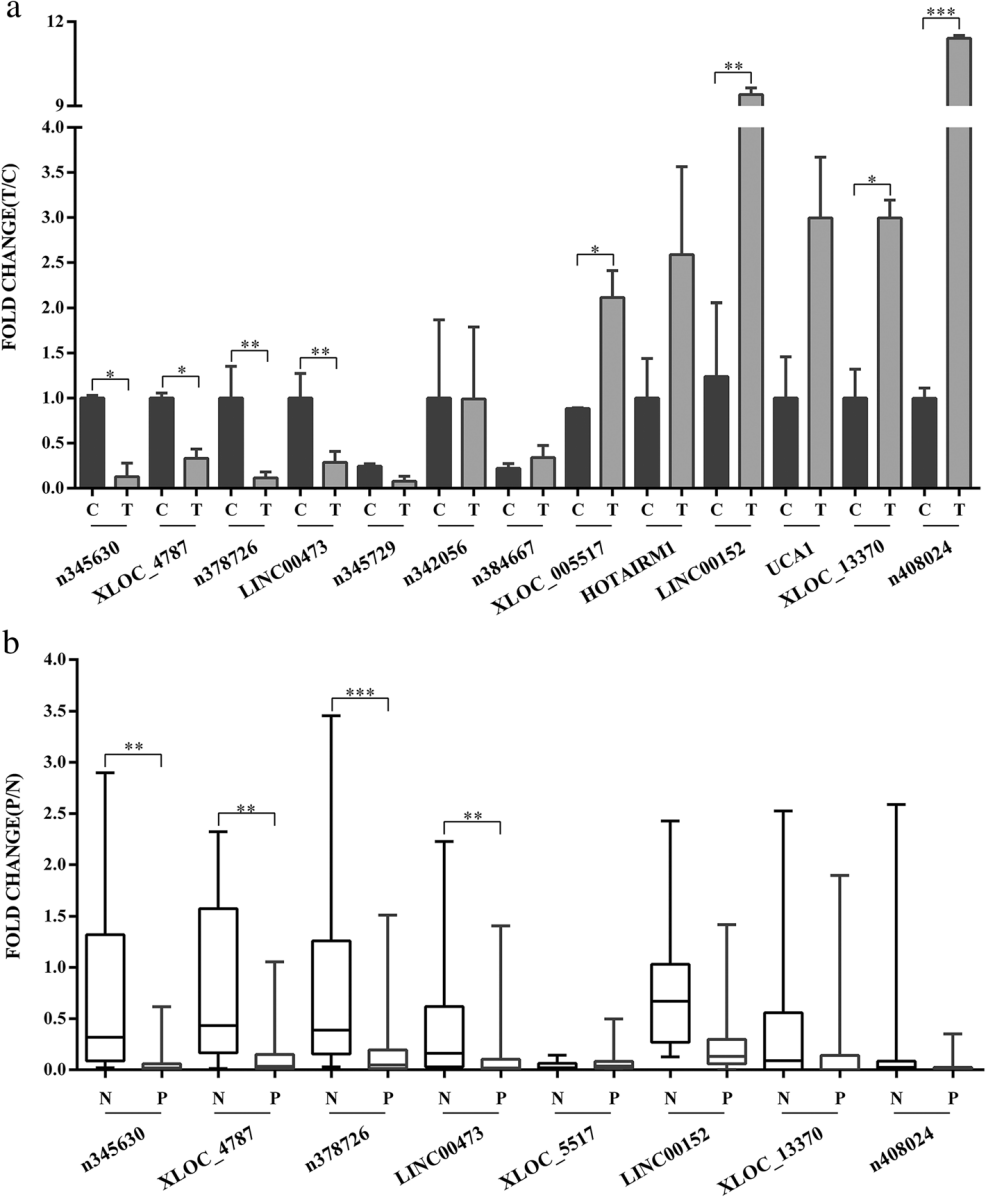
Expression patterns of the candidate lncRNAs in cells and clinical tissue samples. **a**: QRT-PCR validation of 13 candidate lncRNAs relative expression levels in *H.pylori*-infected GES-1 cells compared with controls. “Column T” represents experimental groups, and “column C” represents control groups. **b**: QRT-PCR validation of eight lncRNAs in 156 clinical tissue samples. “Column P” stands for *H.pylori-*positive tissue samples, and “column N stands for *H.pylori*-negative tissue samples. All results were expressed as the means ± SD of three independent experiments with significant *P*-values (*P < 0.05, **P < 0.01, ***P < 0.001)

To confirm the accuracy and repeatability of the microarray data, 13 candidate lncRNAs were selected for validation by qRT-PCR based on their features, such as fold change, adjacent co-expressed mRNAs, and literatures reported. PCR was first carried out to confirm the expression of candidate lncRNAs in GES-1 cells (Additional file 5). The lncRNAsexpression pattern detected by qRT-PCR analysis was the same as that determined by microarray analysis. The changes were statistically difference for only 8 of the 13 lncRNAs (Fig. 4a). We confirmed that n345630, XLOC_004787, n378726, and LINC00473 were suppressed during *H. pylori* infection, whereas the expression of XLOC_005517, LINC00152, XLOC_13370 and n408024 were induced (*P* < 0.05). The 13 candidate lncRNAs were also assessed in other two kinds of gastric cancer cell lines upon *H.pylori* treatment. However, the expression patterns of candidate lncRNAs in BGC-823 and SGC-7901 were very different from those in GES-1 cell lines under the same conditions (Additional file 6).

Then we confirmed the aberrant expression pattern of the 8 candidate lncRNAs in 156 clinical specimens by qRT-PCR. The results indicated that n345630, XLOC_004787, n378726, and LINC00473 were down-regulated (*P* < 0.05) in 67 *H.pylori*–positive specimens compared with 89 negative specimens (Fig. 4b), whereas no significant changes were observed in XLOC_005517,  XLOC_13370 or n408024. Interestingly, there was an opposing expression pattern of LINC00152 in clinical specimens compared with the microarray data (*P* < 0.001).

## Discussion

*H.pylori* possesses numerous factors to successfully colonize the gastric mucosa, influence host immune system, and induce gastric pathology. For the past several decades the molecular mechanisms of *H.pylori* have been widely studied, while the pathogenesis of this agent is still indefinite and most of the involved gene transcriptional regulations remain to be defined. The recent studies on microRNAs (miRNAs) have expanded our insights on *H.pylori* pathogenesis. MiRNAs are a class of non-coding RNAs, short in length, participate in post-transcriptional regulation, and play an important role in multiple biological functions. Aberrantly expressed miRNAs contributed to human diseases and carcinogenesis. Numerous miRNAs have been reported to involve in *H.pylori*-associated gastric pathology by changing the expression of target mRNAs [22, 23]. Zhang et al. reported that miR-21 over-expression was associated with increased cellular proliferation and antiapoptosis in *H.pylori*-positive gastric tissues [24]; miR-146a and miR-155 were specifically involved in the attenuation of the proinflammatory responses against *H.pylori* [25, 26].

LncRNAs are emerging non-coding RNAs in molecular research, longer in length, involved in almost each level of gene expression and regulate diverse functions, such as genome rearrangement, chromatin modification, imprinting, transcription, splicing and translation [27–29]. Increasing discoveries indicated that, like miRNAs, lncRNAs played distinguished roles in pathogenesis and tumorigenesis, and could be novel biomarkers and potential therapeutic targets in diseases [30]. However, only few of lncRNAs were studied to be related to diseases, majority of which are unrevealed and so are those in the *H.pylori*-induced diseases. Thus, we conducted the current study to uncover the role of *H.pylori* in gastric pathological development from a brand new sight of lncRNA. There was only one report exploring the expression profiles of lncRNAs in gastric epithelial cell response to *H.pylori* infection. Liu et al. found that two differentially expressed lncRNAs, XLOC_014388 and XLOC_004122, in *H.pylori*-positive tissues, might be involved in the immune response against *H. pylori* infection [31].

In this study, we used HTA 2.0 microarray to detect the expression profiles of lncRNAs in *H.pylori*-infected GES-1 cells in vitro. We chose gastric epithelial cell line GES-1 for its versatility, non-malignancy and widely used in studying cell signaling cascades response to *H.pylori* infection [32]. Our data showed a set of differentially expressed lncRNAs, including 171 down-regulated and 132 up-regulated lncRNAs in infected cells. Construction of lncRNA-mRNA co-expression network displayed aberrantly expressed lncRNAs/mRNAs which were significantly correlated with their adjacent mRNAs/lncRNAs. From the network of experimental group, we observed that two lncRNAs (n336000 and XLOC_013370) were involved in the most connections (32°) with other transcripts, including 10 target mRNAs (Table 3). As for mRNAs, IER2 was correlated with 23 lncRNAs of the total 34 connections (data not shown). In addition, we found that IER2 and n334184 owned the highest value of |DiffK| in lncRNA-mRNA co-expression network, which indicated that they might play key roles in the development of *H.pylori*–associated disorders and diseases through interaction with many other transcripts (Table 4).

Furthermore, in order to predict the potential functions of lncRNAs, we used GO analysis and KEGG Pathway annotation to investigate the lncRNA co-expressed mRNAs. GO enrichment analysis revealed that the number of genes corresponding to up-regulated mRNAs was larger than that corresponding to down-regulated mRNAs. Pathway annotation showed that there were 54 up-regulated pathways and 40 down-regulated pathways. The significantly enriched up-regulated pathways like HIF-1 signaling pathway, MAPK signaling pathway, cytokine-cytokine receptor interaction, p53 signaling pathway, and Jak-STAT signaling pathway, contained significantly up-regulated mRNAs, such as VEGFA, MMP1, JUN, MYC, EGFR, FGF2, HK2, ICAM1, CSF1, IL1A, etc (Additional file 4). The overexpression of these genes is contributed to promoting cell proliferation, differentiation, metastasis, antiapoptosis, immune responses, and multiple genetic transcriptions. The aberrantly expressed lncRNAs which were co-expressed with these genes in network might have involved in and play collective roles in mediating such processes in *H.pylori* infection.

We initially validated a number of interesting candidate lncRNAs for further study,including 7 down-regulated and 6 up-regulated lncRNAs. We finally found that 8 lncRNAs (n345630, XLOC_004787, n378726, LINC00473, XLOC_005517, LINC00152, XLOC_13370, and n408024) were consistent with microarray results in cell models. There were discrepancies between the results of aberrantly expressed lncRNAs detected in different cell models (SGC-7901 and BGC-823). SGC-7901 and BGC-823 cell lines are from human gastric adenocarcinoma; their cellular responses against *H.pylori* were very different from those in normal gastric mucosal epithelial cells (showed in Additional file 6). So we concluded that the lncRNA expression profiles of those two cancer cell lines were also very different. The lncRNAs, n345630, XLOC_004787, n378726, and LINC00473 were exhibited down-regulation in 67 *H.pylori*–positive mucosa specimens compared with negative specimens. Increased expression of LINC00152 has been reported in gastric cancer and was involved in cell proliferation [33, 34]. At first we supposed that LINC00152 might be involved in pathogenesis of *H.pylori* associated digestive diseases. The qRT-PCR validation of LINC00152 expression pattern in *H.pylori*–infected cells was confirmed to be consistent with microarray data (2.11-fold change). However, when it came to the gastric mucosa tissues, we found reversed expression pattern (*P* < 0.001) out of expectation.

## Conclusions

In this preliminary study, we identified a subset of aberrantly expressed lncRNAs in *H.pylori*-infected models and gastric mucosa tissues. Our data suggested that these novel lncRNAs might contribute to the pathological responses and development of *H.pylori* related disorders and diseases. However, there are still a lot of problems remained to be addressed. Further mechanism studies of these versatile molecules are most important required, which will broaden our understanding of pathogenesis and provide new approaches to the diagnosis and therapies of *H.pylori* infection.

## Additional files

Additional file 1: Table S1. Clinical specimens information. (XLSX 15 kb) Additional file 2: Table S2. Aberrantly expressed lncRNAs and mRNAs in GES-1 cells infected by *H.pylori*. (XLSX 621 kb) Additional file 3: Table S3. GO analysis of aberrantly expressed mRNAs. (XLSX 24 kb) Additional file 4: Table S4. KEGG Pathway analysis of aberrantly expressed mRNAs. (XLSX 32 kb) Additional file 5: Figure S1. Confirmation of expression of 13 candidate lncRNAs in GES-1 cells. (TIFF 9954 kb) Additional file 6: Figure S2. Expression patterns of the candidate lncRNAs in BGC-823 and SGC-7901 cells. (TIFF 2680 kb)

## Abbreviations

LncRNAs: Long non-coding RNAs
*H.pylori*: *Helicobacter pylori*
FDR: False discovery rate
GO: Gene Ontology
KEGG: Kyoto Encyclopedia of Genes and Genomes
