# Supplementary figures and images for: Microarray analysis of Long non-coding RNA expression profiles in human gastric cells and tissues with Helicobacter pylori Infection
Source: BMC Med Genomics. 2015 Dec 21;8:84. doi: 10.1186/s12920-015-0159-0 (PMC4687289; doi:10.1186/s12920-015-0159-0)

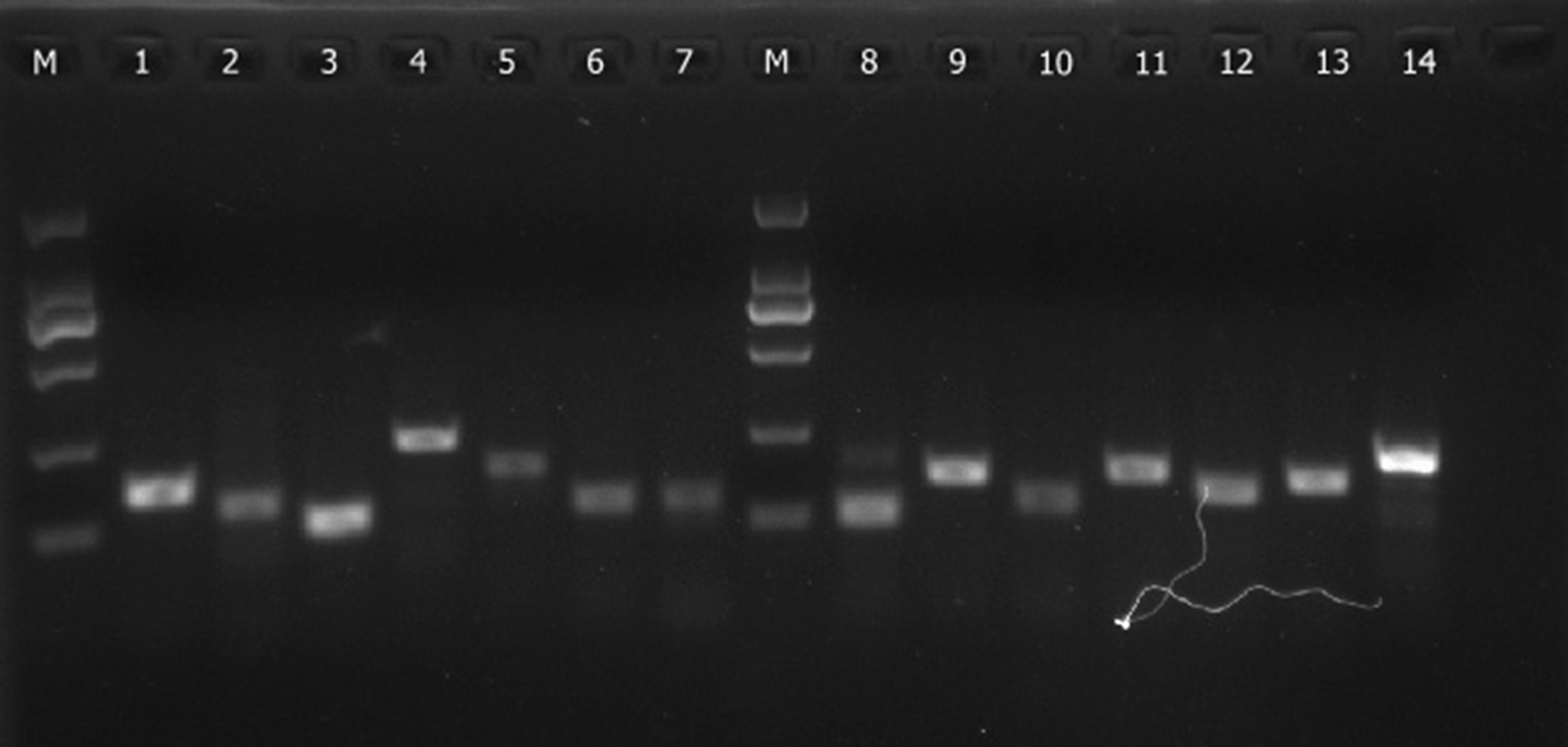

Supplement: Additional file 5: Figure S1. — Confirmation of expression of 13 candidate lncRNAs in GES-1 cells. (TIFF 9954 kb) [file 12920_2015_159_MOESM5_ESM.tiff]

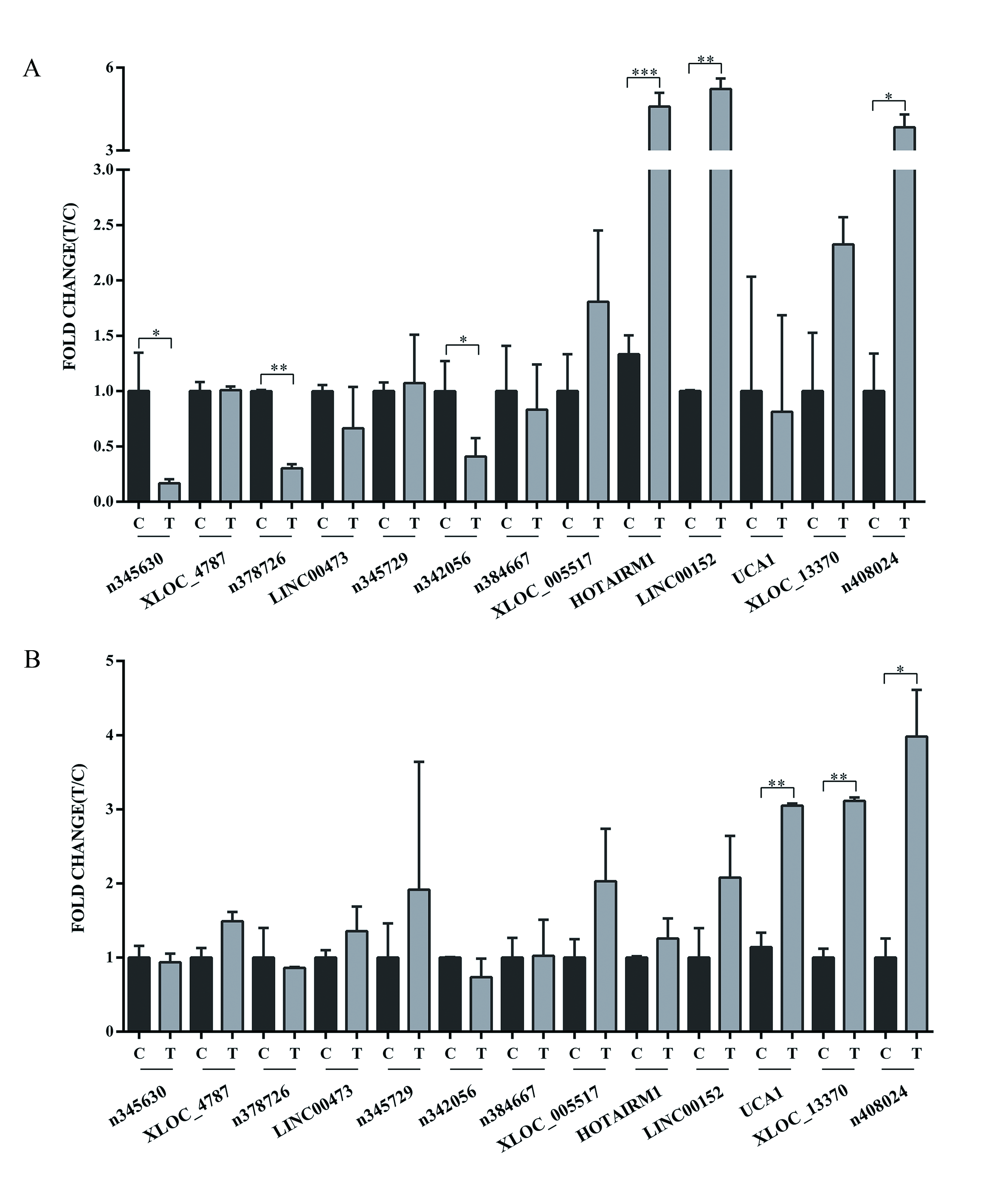

Supplement: Additional file 6: Figure S2. — Expression patterns of the candidate lncRNAs in BGC-823 and SGC-7901 cells. (TIFF 2680 kb) [file 12920_2015_159_MOESM6_ESM.tiff]
